# MiR-222 in Cardiovascular Diseases: Physiology and Pathology

**DOI:** 10.1155/2017/4962426

**Published:** 2017-01-03

**Authors:** Shengguang Ding, Haitao Huang, Yiming Xu, Hao Zhu, Chongjun Zhong

**Affiliations:** Department of Thoracic and Cardiovascular Surgery, The Second Affiliated Hospital of Nantong University, Nantong 226001, China

## Abstract

MicroRNAs (miRNAs and miRs) are endogenous 19–22 nucleotide, small noncoding RNAs with highly conservative and tissue specific expression. They can negatively modulate target gene expressions through decreasing transcription or posttranscriptional inducing mRNA decay. Increasing evidence suggests that deregulated miRNAs play an important role in the genesis of cardiovascular diseases. Additionally, circulating miRNAs can be biomarkers for cardiovascular diseases. MiR-222 has been reported to play important roles in a variety of physiological and pathological processes in the heart. Here we reviewed the recent studies about the roles of miR-222 in cardiovascular diseases. MiR-222 may be a potential cardiovascular biomarker and a new therapeutic target in cardiovascular diseases.

## 1. Introduction

Cardiovascular disease is a predominant cause of morbidity and mortality in the world [1]. The number of patients suffering from cardiovascular disease is growing larger and larger. The major categories of cardiovascular disease include disease of the blood vessels and the myocardium. The contemporary view thinks that most cardiovascular diseases resulted from a complex dysregulation of genetics and environmental factors. Also there are many molecular components that participate in this process, including noncoding RNAs.

MicroRNAs (miRNAs and miRs) are endogenous 19–22 nucleotide, small noncoding RNAs with highly conservative and tissue specific expression. miRNAs can modulate mRNA levels through decreasing transcription or posttranscription induced mRNA decay [2]. Since the first discovery of miRNAs in 1993, they have been found in many species and could participate in various physiological and pathological processes [3–6]. So far, there are over 1000 miRNAs that have been identified, among which at least 200 miRNAs are consistently expressed in the cardiovascular system [7]. miRNAs can regulate cardiomyocytes hypertrophy, senescence, apoptosis, autophagy, and metabolism. Changes of miRNAs have been found to participate in the genesis of many diseases including cardiovascular diseases [8].

miR-222, firstly discovered in human umbilical vein endothelial cells (HUVECs), has been reported to play important roles in epithelial tumors evidenced by its frequently increased expressions in epithelial tumors [9]. Reduction of miR-222 could inhibit cell proliferation and induce mitochondrial-mediated apoptosis through directly targeting the p53 upregulated modulator of apoptosis (PUMA) in breast cancer [10]. Its function on proliferation has also been confirmed in glioblastomas, thyroid papillary cancer, breast cancer, pancreatic cancer, hepatocellular carcinoma, and lung cancer [11–15]. On the other hand, miR-222 can play tumor-suppressive roles through the downregulation of c-kit in erythroleukemia cells. Apart from its role in cancer progress, miR-222 has been found to participate in many physiological and pathological processes in the cardiovascular system (Table 1). Here we reviewed the recent studies about the roles of miR-222 in cardiovascular diseases. MiR-222 may be a potential cardiovascular biomarker and a new therapeutic target in cardiovascular diseases.

## 2. MiR-222 Regulates Physiological Function

**Figure 1:**
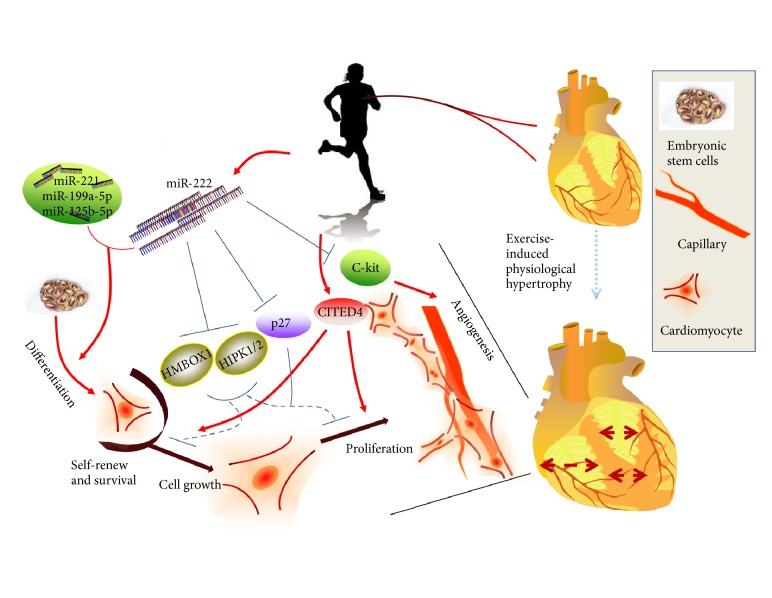
Multiple physiological functions of miR-222 (miR-222 has been found to participate in multiple physiological functions in cardiovascular system. In cardiac myocyte, miR-222 could promote cardiomyocytes growth, proliferation, and survival through directly targeting P27, HIPK-1, HIPK-2, and CITED-4 in traditional exercise pathway. In stem cell, miR-222 could promote CSCs transformation. In umbilical vein endothelial cells, miR-222 could exert angiogenesis function by targeting c-Kit).

The role of miR-222 in regulating physiological process is on a cutting edge of studies (Figure 1).

### 2.1. MiR-222 Regulates Physiological Function in Cardiomyocytes

Physical exercise can induce cardiac growth mainly via hypertrophy and renewal of cardiomyocytes [16]. Unlike pathological hypertrophy, which is related to myocardial structural disorder and cardiac dysfunction, physiological hypertrophy is characterized by normal cardiac structure and normal or improved cardiac function [28]. MiR-222 expression levels were found to be commonly increased in two distinct models of exercise, namely, voluntary wheel running and a ramp swimming exercise model as well as the exercise rehabilitation after heart failure in human. MiR-222 was able to promote cardiomyocytes hypertrophy, proliferation, and survival through directly targeting p27, HIPK-1, HIPK-2, and HMBOX1 [17].

### 2.2. MiR-222 Regulates Physiological Function in Cardiac Stem Cells

Heart has limited regenerative capacity, which might be based on cardiomyocyte division and cardiac stem and progenitor cell activation [29]. Cardiac stem cells (CSCs) are self-renewing, clonogenic, and multipotent, and they can differentiate to mature cardiomyocytes and improve the function and regeneration of the cardiovascular system [30]. CSCs can be activated by physical exercise training [18]. Interestingly, it has been found that the upregulation of miR-222 induced by coculturing human embryonic-stem cell-derived cardiomyocytes (m/hESC-CMs) with endothelial cells could increase and promote CSCs transformation to cardiomyocyte [18].

### 2.3. MiR-222 Regulates Physiological Function in Human Umbilical Vein Endothelial Cells

Human umbilical vein endothelial cells (HUVECs) have unique ability to form capillary-like structures in response to some stimuli. MiR-222 has been reported to exert angiogenesis function through modulating HUVECs angiogenic activity by targeting c-Kit [31, 32].

### 2.4. Sex-Specific Expression of miR-222

There are differences between men and women in cardiovascular diseases incidence, while studies show that males are more likely to suffer from heart attacks than females [33, 34]. MiR-222 are encoded on the X chromosome in mouse, rat, human and have sex-specific expression. Studies have indicated that miR-222 was specifically decreased in mature female mouse hearts as compared with male mouse hearts [31, 35].

## 3. MiR-222 Regulates Pathological Function

**Figure 2:**
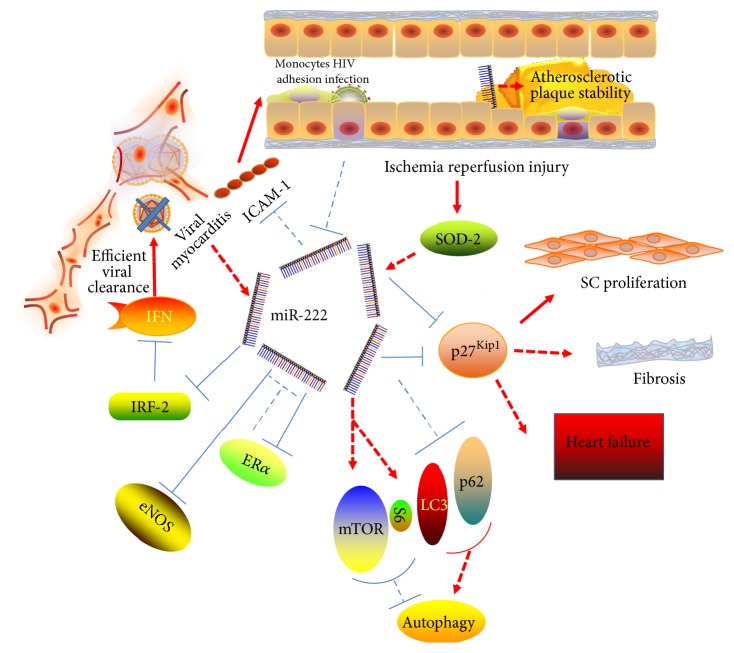
Multiple pathological functions of miR-222 (miR-222 has been found to participate in multiple pathological functions in cardiovascular system. In myocardium, miR-222 could (1) promote cardiomyocyte proliferation and reduce cardiomyocyte apoptosis through P27 after ischemic injury; (2) inhibit autophagy through mTOR; (3) regulate blood vessels remolding through c-Kit and eNOS; (4) regulate ICAM-1 and IRF-2 to inhibit inflammation. In blood vessels, miR-222 could (1) stable the plaque and suppress the inflammation and (2) inhibited the proliferation of vascular smooth muscle by targeting p27).

Unraveling the role of miR-222 in regulating cardiac pathological function may foster new therapeutic targets for cardiovascular diseases (Figure 2).

### 3.1. MiR-222 Regulates Pathological Function in Myocardium

#### 3.1.1. Cardiac Ischemia Reperfusion Injury

Myocardial ischemic reperfusion is a complex process involving numerous mechanisms including reactive oxygen species (ROS) overload, inflammation and calcium overload, energy metabolism dysfunction, and mitochondrial permeability transition pore (mPTP) opening [36–38]. MiR-222 has been reported to be able to protect against cardiac dysfunction after ischemic injury. MiR-222 can promote cardiomyocyte proliferation and reduce cardiomyocyte apoptosis through P27. In addition, miR-222 overexpression mice have well-preserved cardiac function and reduced cardiac fibrosis when subjected to cardiac ischemia reperfusion [17].

#### 3.1.2. Heart Failure

Heart failure is the terminal outcome of the majority of cardiovascular diseases, and it seriously reduces the quality of life. A significant inhibition of autophagy in Tg-miR-222 mice after heart failure was observed, which was through mTOR, a negative regulator of autophagy [19]. Inhibition of autophagy induced by miR-222 may cause accumulation of protein and organelles injury, even the impairment of cardiac function. Angiogenesis has been proposed as a promising therapy for ischemia heart disease and heart failure. miR-221/222 family seemed to inhibit angiogenesis [21]. MiR-222 was significantly decreased in endothelial cells (ECs) when cultured for 24 h with HDL from chronic heart failure (CHF) patients compared to healthy control. The downregulation of miR-222 may be a compensatory mechanism of ECs to counteract cardiovascular adverse events [39].

#### 3.1.3. Viral Myocarditis

Cardiac inflammation is an important cause of dilated cardiomyopathy and heart failure. In young healthy adults, it can cause sudden death. Viral myocarditis is one of cardiac inflammation diseases. MiR-222 has been reported to be able to orchestrate the antiviral and anti-inflammatory response through downregulation of IRF-2 [23]. Inhibition of miR-222 would increase the risk of cardiac injury. HIV-infected cardiomyopathies is another kind of inflammation diseases [22, 40]. MiR-222 can regulate cell adhesion molecules ICAM-1 translation directly or indirectly (through IFN-*γ*) to inhibit inflammation [22, 41].

#### 3.1.4. Congenital Heart Disease

Tetralogy of Fallot (TOF) is one of the most common congenital heart malformations in children [42]. miR-222 was found to display a high expression level in right ventricular outflow tract (RVOT) tissues compared with controls. Cardiac myocyte proliferation and differentiation is a key event in heart development. Further functional analysis showed that overexpression of miR-222 promoted cell proliferation and regulated cell differentiation by inhibiting the expression of the cardiomyocyte marker genes during the cardiomyogenic differentiation [25]. In another congenital heart disease, ventricular septal defect, the decreased expression of miR-222 also indicated its important role in heart development [20].

### 3.2. MiR-222 Regulates Pathological Function in Blood Vessels

#### 3.2.1. Atherosclerosis

During the genesis of atherosclerosis, there are various molecules and cellular components that can make atherosclerotic plaque vulnerable and even rupture [43]. Many studies show that miRNAs also participate in this process [44]. MiR-222 derived from ECs may play its protective role by blocking intraplaque neovascularization and suppressing the inflammatory activation of ECs, without enhancing the proliferation of ECs [45, 46].

#### 3.2.2. Peripheral Arterial Disease

Smooth muscle cells (SMCs) constitute the medial layer of arteries and regulate the vascular tone via their contractile apparatus [27]. MiR-222 was reported to take part in the development of neointima and promotes neointima formation after vascular injury by enhancing the proliferation of SMCs. Furthermore, in the peripheral artery disease (PAD) caused by atherosclerosis or inflammation of the peripheral arteries, studies have showed that miR-222 also inhibited the proliferation of vascular smooth muscle cell by targeting p27 [45] to stable the plaque [24] and promoted skeletal muscle regeneration after ischemia. Besides that, under the administration of superoxide dismutase-2 (SOD-2), miR-222 plays its protective role against peripheral artery disease by regulating p57 expression [26] but not P27.

## 4. Conclusions

In conclusion, miR-222 controls many cardiac physiological functions and its deregulation has been implicated in many cardiovascular diseases. Targeting miR-222 might be a promising therapeutic target for cardiovascular diseases.

## Figures and Tables

**Table 1:** Summary of physiological and pathological functions of miR-222 in heart.

	Cardiac parameter	Model		Effects of miR-222	References
		In vitro	In vivo		
Physiological function	Cardiomyocytes proliferation	Neonatal rat ventricular cardiomyocytes Adult mice cardiomyocytes Adult mice noncardiomyocytes	C57BL/6J, exercise, and cardiac ischemia reperfusion surgery	Cardiomyocytes growth, proliferation, and survival in vitro ↑ Necessary for exercise-induced cardiac growth	[13, 17]
	Cardiac stem/progenitor cells differentiation	Mouse ESCs Human ESCs	—	Sarcomere alignment and calcium handling ↑ Resting membrane potential ↓ cardiomyocytes maturation markers ↑	[18]

Pathological function	Ischemia reperfusion injury	—	miR-222 overexpression mice, cardiac ischemia reperfusion surgery	Protecting against cardiac dysfunction after I/R	[17]
	Heart failure	—	miR-222 overexpression mice, cardiac-specific	Inducing heart failure Inhibiting autophagy	[19]
		—	Human	miR-222 ↓ in HF patients with left ventricular assist devices	[20]
		Human aortic endothelia cells	—	LDL from HF patients ↓ miR-222	[21]
	Inflammation	HUVECs	—	HIV Tat protein ↓ miR-222 Involved in inflammatory pathway	[22]
		Adult mouse cardiomyocytes, nRCMs, MCECs, and nRCFs	C57BL/6J, C3H, viral myocarditis	Cardiac viral infection ↑ IFN through miR-222 emerging efficient viral clearance	[23]
	Atherosclerosis	—	Human	miR-222 ↓ in atherosclerotic plaque shoulder related to plaque rupture	[24]
	Tetralogy of Fallot	Primary embryonic mouse cardiomyocytes; P19 Cell Line	Human	↑ In heart tissue of patients ↑ migration, proliferation in embryonic mouse cardiomyocytes ↓ Cardiomyogenic differentiation of P19 cells	[25]
	Ventricular septal defect	—	Human	Low level in patients with ventricular septal defect	[20]
	Peripheral artery disease	—	C57BL/6J, Hindlimb ischemia surgery	↑ Skeletal muscle regeneration after hindlimb ischemia	[26]
	Artery damage	—	ApoE Knockout mice, wire-induced artery injury	↑ During neointima formation	[27]
